# Bilateral Sternalis Muscle With the Absence of Unilateral Sternocostal Part of the Pectoralis Major and Variation of Pectoralis Minor Muscles

**DOI:** 10.7759/cureus.41653

**Published:** 2023-07-10

**Authors:** Devendra Shekhawat, Dinesh Kumar, R. Shane Tubbs

**Affiliations:** 1 Anatomy, Maulana Azad Medical College, New Delhi, IND; 2 Anatomical Sciences, St. George's University, St. George's, GRD; 3 Neurosurgery and Structural & Cellular Biology, Tulane University School of Medicine, New Orleans, USA

**Keywords:** rectus sternalis, anatomic variations, sterno-pectoral region muscles, pectoralis minor, pectoralis major

## Abstract

This study aims to report a 57-year-old male cadaver with a rare muscular variation of the sterno-pectoral region. An unusual sternalis muscle was observed on both sides, arising from the external oblique muscle aponeurosis. The fibers converged upwards and medially in a curved course, producing a bundle 99.50 mm long on the right side and 74.60 mm on the left. The muscles on both sides were supplied by the second, third, and fourth intercostal nerves. In the right pectoralis major (PM) muscle, the sternocostal head was completely absent, and the clavicular head arose from the medial two-thirds of the clavicle, whereas abdominal fibers arose from the aponeurosis of the external abdominal oblique muscle and ran upward and laterally and joined the clavicular fibers with a wide triangular gap. On the left side, there was an anatomically normal PM muscle. The origin of the pectoralis minor was unusually high on both sides. The morphological variations of sterno-pectoral musculature have significant implications for clinical practice, which allows more precise surgical or radiological outcomes. Clinicoradiological evaluation of these variations is important to achieve appropriate dissection planes during chest wall surgery.

## Introduction

The sterno-pectoral region is clinically important for physicians, radiologists, and surgeons. The sternalis muscle is an aberrant muscle of the anterior chest wall. Cadaveric investigations revealed that the overall prevalence of this muscle is 5.96% in adults, based on 76 studies, which was higher than other modalities of investigation [1]. The predetermination of anatomical anomalies of the anterior chest wall through imaging can enable it to be used as a muscular flap in reconstructive or plastic surgery of the head and neck [2]. Here, we report bilateral sternalis muscle with the absence of a unilateral sternal portion of the sternocostal part of the pectoralis major (PM) and a high origin of pectoralis minor (PMi). To the best of our knowledge, this is a unique variation.

## Case presentation

Morpho-muscular variations were observed in the sterno-pectoral region in one 57-year-old adult male among the 75 cadavers dissected over the time of 16 years. There was no history of trauma or surgery in this region.

Rectus sternalis

**Figure 1:**
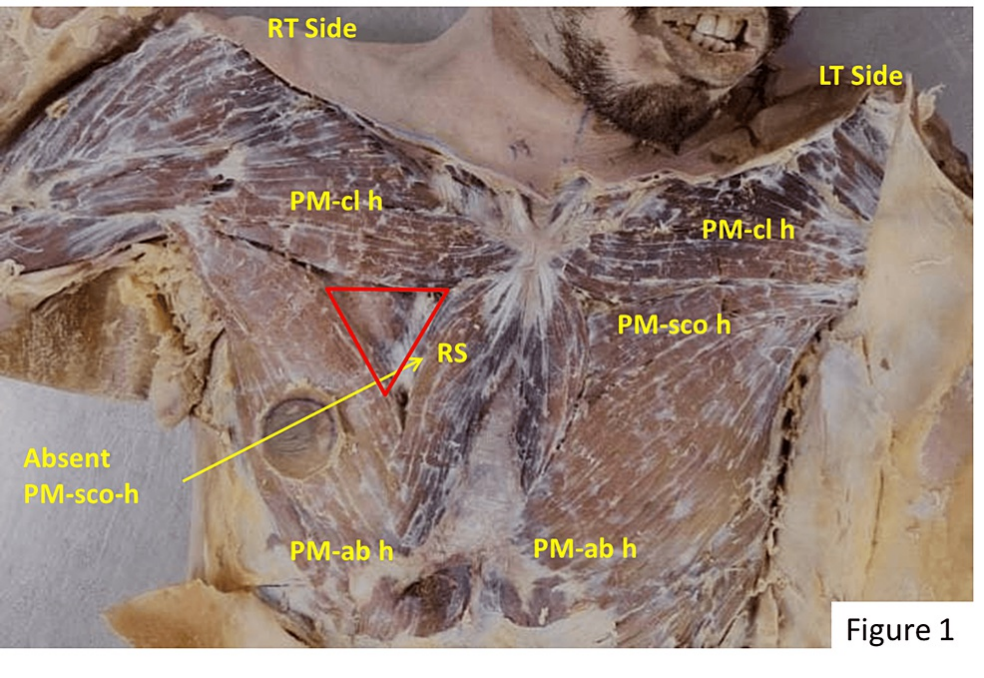
Anterior chest wall of the cadaver after removing the skin and fascia. PM-cl h: pectoralis major clavicular head; PM-sco h: pectoralis major sternocostal head; PM-ab h: pectoralis major abdominal head; RS: rectus sternalis.

Strap-like, flattened sternalis muscles were observed bilaterally on either side of the sternum, lying superficial to the PM and its fascia (Figure 1). The lower fibers of the rectus sternalis (RS) on both sides arose as fleshy fibers from the right external abdominal oblique muscle aponeurosis. These fibers converged upwards and medially in a curved course, making a bundle with a total length of 99.50 mm on the right side and 74.60 mm on the left. The muscle bundles on both sides converged at the manubrium sterni, where they intermingled with each other and with fibers of the clavicular head of the right PM. The second, third, and fourth intercostal nerves supplied both sides.

Pectoralis major

On the right side, the origin of the PM muscle showed anatomical variation. The clavicular head (PM-cl h) arose from the anterior surface of the medial two-thirds of the clavicle but the sternocostal (PM-sco h) head was completely absent (Figure 1). Both these muscle fibers ran horizontally and laterally to make a bundle with a maximum thickness of 69.53 mm. The sternalis on the right side ran upward and laterally and joined the clavicular part of the PM muscle. There was a wide triangular gap in the PM on the right side. The lower bundle of PM fibers (abdominal part - PM-ab h) originated from the external abdominal oblique, reached the anterior axillary fold, and was inserted on the lateral lip of the bicipital groove. There was a 66.71 mm separation between the clavicular and lower fibers at their respective origins. The vertical height of the anterior wall of the axilla was 10 mm less than on the left side. There was an anatomically normal PM muscle on the left. Both muscles were supplied by the medial and lateral pectoral nerves.

Pectoralis minimus and pectoralis minor

**Figure 2:**
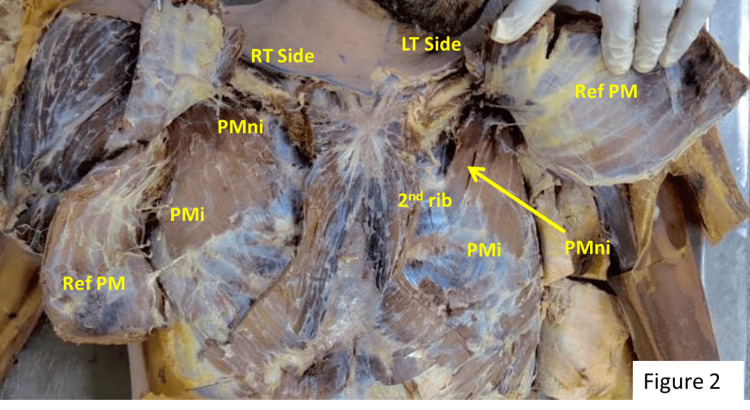
Anterior chest wall of the cadaver after reflected pectoralis major. Ref PM: reflected pectoralis major; PMi: pectoralis minor; PMni: pectoralis minimus muscle.

The pectoralis minimus (PMni) originated from the second rib and its costal cartilage. After taking of origin, the inferior fibers were merged with the upper border of the PMi muscle, which originated from the third to fifth ribs and costal cartilage. Both muscle fibers made a single belly, which ran laterally and upward to get inserted into the coracoid process (Figure 2).

Anatomical variant triangle in the right pectoral region

A prominent triangle was formed in the right pectoral region, bounded medially by the lateral border of the right sternalis, which was 62.09 mm long, superolaterally by the lower border of the clavicular head of the PM muscle, which was 72.54 mm long, and inferolaterally by the upper border of the abdominal part of the PM muscle. The superolateral and inferolateral borders met at the apex, 7.12 mm above the anterior axillary fold. The roof of the triangle was formed by skin and superficial fascia along with the covering of pectoral fascia. The floor was formed by the second, third, and fourth ribs and the intercostal muscles on the medial side, and the origin of the PMi on the lateral side (Figure 1).

## Discussion

The sternalis muscle is known by many names, such as episternalis, parasternalis, rectus thoracicus, and sternalis superficialis [3]. Cadaveric investigations reveal that the sternalis muscle has an overall prevalence of around 7.8% in the general population, with the highest (11.5%) in the Asian population and the lowest (4.4%) in the European population [4].

Its prevalence is not well documented in India. Few studies of the Indian population include those by Vijaianand et al. [5] and Katara et al. [3], who reported prevalence rates of 5.4% (two of 37 cadavers) and 3.3% (one of 30 cadavers), respectively. In our study of north Indian cadavers, the prevalence was 1.33% (one of 75 cadavers) and this case was accompanied by other abnormalities in the pectoral region musculature. To the best of our knowledge, this type of case has not been reported previously.

After extensive research on the sternalis muscle, Snosek et al. stated that its anomalies could be attributed to disturbance of the normal process of PM muscle development, resulting in variations such as PMni, pectoralis tertius, infraclavicular, and chondroepitrochlearis in the pectoral region. The sternalis muscle is therefore associated with atypical development of the PM or deficiencies in its medial aspect [4].

The sternalis muscle has never been related to any clinical symptoms. However, it can cause alterations in the electrocardiogram or confuse routine mammography [6]. Therefore, it can lead to misdiagnosis, giving the false impression of an irregular structure, often mistaken for a wide range of benign and malignant chest walls or breast lesions such as breast carcinoma or hematoma. Imaging techniques such as multidetector CT and MRI, and modern techniques such as three-dimensional volume reconstruction from CT or MRI studies, can help to preclude the diagnostic dilemma [7]. The sternalis muscle can also cause breast or chest asymmetry or deviation of the ipsilateral nipple-areola complex [8], especially when it co-exists with other PM defects [9].

During the interpretation of mammograms, the sterno-pectoral muscles should be considered cautiously because there is a potential blind spot on the median side of the mammogram. Craniocaudal projection with adequate traction of the breast can help to include obscure portions. Awareness of this aberrant structure is important in anterior thoracic wall surgical dissection, particularly in breast and cardiothoracic surgery. In augmentation mammoplasty, the muscle can interfere with the dissection of the submuscular pocket, causing an unexpected outcome. However, owing to its functional insignificance, the sternalis can be used as a muscle flap in the anterior chest wall, head and neck, and breast reconstruction [10].

The PM has an important relationship with the chest wall and breast. It can be considered one of the key anatomical structures in plastic and reconstructive surgery [11]. Its importance in orthopedic surgery includes, inter alia, the deltopectoral approach to the repair of PM injuries. In our case report, this muscle lacked a sternal head and had a triangular gap. This type of variation has paramount significance for cardiothoracic and plastic surgeons.

Bala et al. (2014) reported a case showing that both muscles, the PM and sternalis, developed from a single muscle mass, and further suggested that the sternalis is an upper limb muscle. We agree with the authors, and, in our case, the PMi muscle had a higher origin [12]. Our aim is to report variations in sterno-pectoral muscles, such as the rectus sternalis, PM, and PMi, which will help clinicians and surgeons make diagnoses related to compression of the axillary artery and brachial plexus.

There are many hypotheses about the origin of the muscle. It is either claimed as a remnant of the panniculus carnosus, a vestige of the cuticular muscle of mammals, presenting as an axillary arch, or as a part of a ventral, longitudinal column of muscles arising at the ventral tips of the hypomeres. Cunningham declared that aberrant, displaced, and rotated segments of the pectoralis muscle mass form the sternalis [13]. The rectus sternalis develops either from the rectus abdominis sheath or from the PM owing to a defect in muscle patterning. In the latter case, the defective precursor migration of the prepectoral mass that gives rise to the PM and PMi muscles can also contribute to the development of the sternalis muscle, while mechanical disturbances can lead to atypical clockwise rotation of the muscle fibers [14]. Alfred Poland described the “deficiency of pectoral muscles” as deficient sternal and costal portions of the PM muscle and absence of PMi muscle in addition to brachysyndactyly of the ipsilateral hand, underdeveloped serratus anterior muscle, and remarkably small thoracic vessels [15,16]. This case report also shows a deficiency of the sternocostal head and a wide triangular gap on the right side with a high origin of the PMi muscle. Clinicians should be aware of such a gap so that it is not misinterpreted in imaging studies or during physical examination of the patient. Additionally, in invasive procedures, e.g., central venous line placement, clinicians should be mindful of such a gap as deeper-lying structures will be near the surface of the patient.

## Conclusions

In conclusion, the morphological variations of sterno-pectoral musculature have significant implications for clinical practice, which allows more precise surgical or radiological outcomes. It is important to have a clinicoradiological evaluation of these variations to achieve appropriate dissection planes during, for example, chest wall surgery. We identified a sternalis muscle in association with a gap between parts of the ipsilateral PM. Knowledge of these variants will avoid misdiagnosis.
